# Evaluation of azacitidine and entinostat as sensitization agents to cytotoxic chemotherapy in preclinical models of non-small cell lung cancer

**DOI:** 10.18632/oncotarget.2695

**Published:** 2014-11-25

**Authors:** Frank P. Vendetti, Michael Topper, Peng Huang, Irina Dobromilskaya, Hariharan Easwaran, John Wrangle, Stephen B. Baylin, J. T. Poirier, Charles M. Rudin

**Affiliations:** ^1^ The Sidney Kimmel Comprehensive Cancer Center, Johns Hopkins University, Baltimore, MD; ^2^ Memorial Sloan Kettering Cancer Center, NY 10065, New York

**Keywords:** epigenetic, azacitidine, entinostat, priming, chemosensitivity, non-small cell lung cancer (NSCLC)

## Abstract

Recent clinical data in lung cancer suggests that epigenetically targeted therapy may selectively enhance chemotherapeutic sensitivity. There have been few if any studies rigorously evaluating this hypothesized priming effect. Here we describe a series of investigations testing whether epigenetic priming with azacitidine and entinostat increases sensitivity of NSCLC to cytotoxic agents.

We noted no differences in chemosensitivity following treatment with epigenetic therapy in *in vitro* assays of viability and colony growth. Using cell line and patient-derived xenograft (PDX) models, we also observed no change in responsiveness to cisplatin *in vivo*. In select models, we noted differential responses to irinotecan treatment *in vivo*. *In vitro* epigenetic therapy prior to tumor implantation abrogated response of H460 xenografts to irinotecan. Conversely, *in vitro* epigenetic therapy appeared to sensitize A549 xenografts (tumor growth inhibition 51%, vs. 22% in mock-pretreated control). *In vivo* epigenetic therapy enhanced the response of adenocarcinoma PDX to irinotecan.

Taken together, these data do not support broadly applicable epigenetic priming in NSCLC. Priming effects may be context-specific, dependent on both tumor and host factors. Further preclinical study is necessary to determine whether, and in which contexts, priming with epigenetic therapy has potential to enhance chemotherapeutic efficacy in NSCLC patients.

## INTRODUCTION

Non-small cell lung cancers (NSCLC) account for approximately 80% of all cases of lung cancer, a disease which remains the leading cause of cancer-related mortality worldwide [1]. The utility of existing targeted therapies for NSCLC remains limited due to the lack of targetable oncogenic drivers in many patients, acquired resistance, and disease recurrence [2–7]. As a result, NSCLC is still frequently treated with conventional cytotoxic chemotherapy, typically a platinum agent in combination with a taxane or gemcitabine in first line therapy. This approach is hampered by limited efficacy, high toxicity, resistance, and recurrence of disease, which highlights the need for more effective treatments.

There is substantial evidence (reviewed elsewhere) that epigenetic dysregulation, including silencing of tumors suppressor genes, is intimately involved in lung carcinogenesis [8–10]. We recently explored the potential efficacy of a combinatorial epigenetic therapy strategy for the treatment of recurrent metastatic NSCLC, using the demethylating agent, azacitidine (Aza), and the class I specific histone deacetylase inhibitor (HDI), entinostat [11]. A small number of patients (2/34) exhibited objective responses to combination epigenetic therapy alone, and 10 others experienced disease stabilization. A surprising observation from this trial was that several patients, including patients with rapid progressive disease while on epigenetic therapy, responded to their next chemotherapy regimen (Fig. S1), despite a median of three prior therapies for this patient population. Two of these patients survived 44 and 52 months after cessation of epigenetic therapy despite receiving only one subsequent therapy [11]. A similar scenario was previously observed in a pilot phase I/II study of the demethylating agent decitabine in stage IV NSCLC patients, with one patient receiving chemotherapy aprroximately six months after decitabine, and surviving 81 months [12]. These observations, although representing a small sample size, suggest the potential for epigenetic therapy to sensitize NSCLC to subsequent chemotherapy. Additional supporting evidence for a chemotherapy priming effect comes from recent clinical trials in other solid tumors: two recent reports provide evidence that treatment with a demethylating agent (azacitidine or decitabine) can re-sensitize resistant and refractory ovarian tumors to platinum chemotherapy [13, 14].

Preclinical evidence also implicates epigenetic mechanisms in drug resistance in cancer [15–19]. Several studies demonstrate sensitization of solid cancers to chemotherapy following treatment with epigenetically directed therapies, in association with reactivation of silenced tumor suppressor genes or restoration of tumor suppressor protein expression [15, 17, 20]. Interestingly, one of these studies found that the combination of decitabine and the pan-HDI, belinostat, was more effective than decitabine alone at reactivating the silenced tumor suppressor, *hMLH1*, and re-sensitizing a cisplatin resistant ovarian cancer cell line to cisplatin both *in vitro* and *in vivo* [17]. Another recent paper directly implicated epigenetic alterations, including increased expression of the histone demethylase, JARID1A/KDM5A, and loss of H3K4me2/me3 histone marks, in tolerance of a mutant *EGFR* NSCLC line to EGFR targeted therapy, and demonstrated the ability to prevent or suppress the phenotype through treatment with HDIs [18]. These latter two studies highlight a distinct role for histone deacetylase (HDAC) inhibition, in addition to DNA demethylation, in reversal of epigenetically mediated resistance mechanisms.

Here we test whether priming with single agent or combination epigenetic therapy sensitizes NSCLC to various subsequent chemotherapeutic agents. We include cisplatin, docetaxel, gemcitabine, and vinorelbine, as they are FDA approved for the treatment of NSCLC, as well as irinotecan, which is included in the National Comprehensive Cancer Network (NCCN) Guidelines for the treatment of NSCLC. Although irinotecan is not commonly used in this setting, the observation from our recent NSCLC clinical trial that two patients who received irinotecan following epigenetic therapy achieved stable disease (Fig. S1) [11] warrants its inclusion. In addition, the hsp90 inhibitor, 17-AAG, and the proteasome inhibitor, bortezomib are included to explore sensitization to drug mechanisms distinct from those of DNA damaging agents or anti-mitotics. It has been shown that combining entinostat with 17-AAG synergistically inhibits growth of several NSCLC cell lines, including A549 [21]. In addition, work in breast cancer cell lines demonstrated that HDAC1 maintains the chaperone, hsp90, in a deacetylated state, allowing its association with and preventing proteasomal degradation of DNA methyltransferase 1 (DNMT1) [22]. In this model, HDAC1 inhibition induces hsp90 hyperacetylation, disrupts association of hsp90 with DNMT1, and promotes ubiquitination and degradation of DNMT1 via the proteasome. Since HDAC1 is a target of entinostat, we hypothesized that pretreatment with entinostat may augment sensitivity to 17-AAG. Bortezomib was also of interest with regard to this pathway as a direct inhibitor of proteasomal function.

Using several preclinical models encompassing two of the three most common histological subtypes, we find that the combination of azacitidine and entinostat enhances sensitivity of select NSCLC tumors to irinotecan *in vivo*, while sensitivity to other agents remains largely unaffected both *in vitro* and *in vivo*.

## RESULTS

### Epigenetic therapy does not sensitize NSCLC cell lines to subsequent chemotherapy in acute cytotoxicity assays

We sought to determine whether epigenetic therapy sensitizes NSCLC cell lines to subsequent chemotherapy *in vitro*. We selected the following for our epigenetic priming regimen: 500 nM Aza every 24 hours for 72 hours (days 0 – 3), 50 nM entinostat for 24 hours (days 2 – 3), combination, or mock treatment, followed by harvesting and replating, and culture for one week in drug free media. This dose and schedule of Aza has been shown to alter gene methylation and expression patterns and exert lasting effects on clonogenic and tumorigenic potential of leukemia, breast, and lung cancer cells [23, 24]. The entinostat dose was selected based on published pharmacokinetic data obtained from patients who received the same 7 mg fixed dose as administered in our recent NSCLC trial [25].

**Figure 1:**
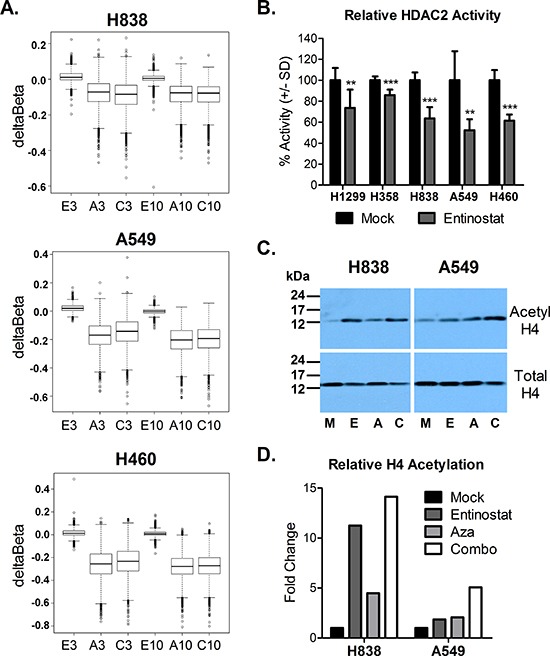
Epigenetic changes associated with azacitidine and entinostat treatment **(A)** Box plots of deltaBeta values depicting promoter region (+/− 1500 bp of transcription start site) demethylation (negative deltaBeta) relative to mock control (probes with Beta >0.5) at day 3 and day 10 following treatment with entinostat (E), Aza (A), or combo (C). **(B)** Percent HDAC2 enzyme activity after 24 h treatment with 50 nM entinostat, relative to mock control. Bars represent the mean of seven replicates +/− standard deviation. Statistical significance by two-tailed, unpaired t-test denoted as follows: **p < 0.01, ***p < 0.001. **(C)** Western blots depicting acetylated histone H4 (lysine 5, 8, 12, 16) and total histone H4 levels at the end of treatment (day 3) with mock (M), entinostat (E), Aza (A), or combo (C). **(D)** Quantified histone H4 acetylation, relative to mock control.

We validated our Aza dosing regimen by assessing DNA methylation using the Illumina Infinium 450 BeadChip. We found that promoter region DNA methylation, including only Infinium probes within 1500 bp (+/−) of the transcription start site, decreased with 72 h Aza or combination treatment in all cell lines (day 3), and demethylation was maintained one week post treatment (day 10) (Fig. 1A and Fig. S2A). To validate our dosing regimen for entinostat, we first assessed live cell activity of HDAC2, one of three relevant isoforms targeted by entinostat [26, 27]. Treatment with 50 nM entinostat suppressed mean HDAC2 activity by 26.5%, 14.2%, 36.4%, 47.7%, and 38.5% in H1299, H358, H838, A549, and H460, respectively (Fig. 1B). We also examined changes in acetylation of histone H4 (lysine residues 5, 8, 12, and 16) relative to total histone H4 levels in four cell lines at the end of treatment with entinostat, Aza, combination, or mock, as described above. We found that entinostat, Aza, and combination treatment increased acetyl-H4 by 11.2-, 4.5-, and 14.1-fold, respectively, in H838, and 1.8-, 2.1, and 5.1-fold, respectively, in A549 (Fig. 1C-D). However, we did not see an increase in acetylation in H1299 and H460 with any treatment (Fig. S2B). These data suggest that the chosen dose may be insufficient, as indicated by the lower degree of HDAC2 inhibition or lack of changes in H4 acetylation, in some cell lines. However, as entinostat has approximately 6-fold greater selectivity for HDAC1 over HDAC2, we anticipate greater inhibition of that isoform [26]. In addition, HDACs, including the targets of entinostat (HDAC1, 2, 3) have many non-histone substrates, and therefore the pleiotropic effects of entinostat and other HDAC inhibitors are likely mediated by more than altered histone acetylation (reviewed elsewhere) [28–31]. Melanoma patients who received a 7 mg fixed oral dose of entinostat in a phase II clinical trial achieved a mean plasma Cmax of 49.15 nM [25]. We therefore chose a dose of 50 nM entinostat for *in vitro* experiments in order to mirror clinically relavent drug exposure.

**Figure 2:**
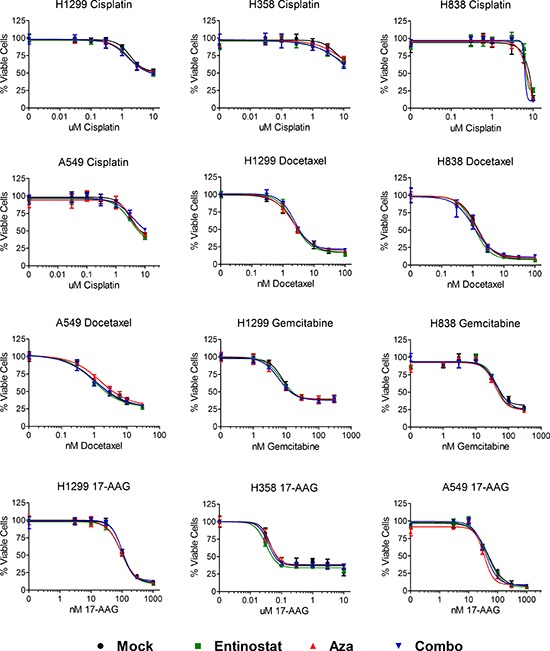
Epigenetic priming does not alter sensitivity of NSCLC cell lines to subsequent chemotherapy Log dose response curves for NSCLC cell lines treated (in triplicate) with cisplatin, docetaxel, gemcitabine, or 17-AAG for 72 hours one week post epigenetic therapy. Individual curves represent the percentage of viable cells (+/− standard deviation) for each epigenetic pretreatment condition normalized to its own untreated control cells, such that the highest values for each pretreatment condition represent 100%, and 0 = 0%. Data shown from representative experiments.

We explored whether cells treated on the above regimens of epigenetic priming or control in days 0 – 3 exhibited increased sensitivity to chemotherapy by seeding cells at equal number on day 9, and treating with chemotherapy for 72 hours beginning on day 10. Following treatment, cell viability was assessed on day 13. Nonlinear regression of background corrected, log-transformed data was performed to obtain IC_50_ values, 95% confidence intervals, and R^2^ for each epigenetic pretreatment condition and chemotherapy tested (Table S1). In cases where a maximal inhibition plateau was not reached and the calculated IC_50_ was ambiguous (e.g. H358 and H838 treated with cisplatin), IC_50_ was considered not determined (ND). Statistical analysis of logIC_50_ and standard error of logIC_50_ via ANOVA with Tukey's multiple comparison test revealed no statistically significant differences in IC_50_ among pretreatment conditions for any evaluable chemotherapy. Log dose response curves from data normalized to untreated controls within each pretreatment group for a given chemotherapy demonstrate minimal differences in chemosensitivity across cell lines, pretreatment conditions, and chemotherapeutic agents tested (Fig. 2 and Fig. S3).

### Epigenetic therapy does not augment inhibition of colony growth by subsequent chemotherapy in NSCLC cell lines

To further test whether epigenetic therapy sensitizes NSCLC to the effects of chemotherapy *in vitro*, we selected sub- or near- IC_50_ doses of select chemotherapeutic agents and assessed the effects of these agents on colony growth with or without pretreatment with epigenetic therapy. Prior work has suggested that exposure to low concentrations of demethylating agents (decitabine or Aza) alone can blunt clonogenic and tumorigenic potential of leukemia and breast cancer, implying depletion of progenitor or tumor initiating cell populations [23]. We hypothesized that pretreatment with epigenetic agents would also attenuate clonogenic capacity of NSCLC. In addition, if epigenetic pretreatment sensitizes clonogenic NSCLC cells to chemotherapeutic agents, we would predict a greater attenuation of clonogenic capacity following chemotherapy than that achieved with epigenetic therapy alone.

**Figure 3:**
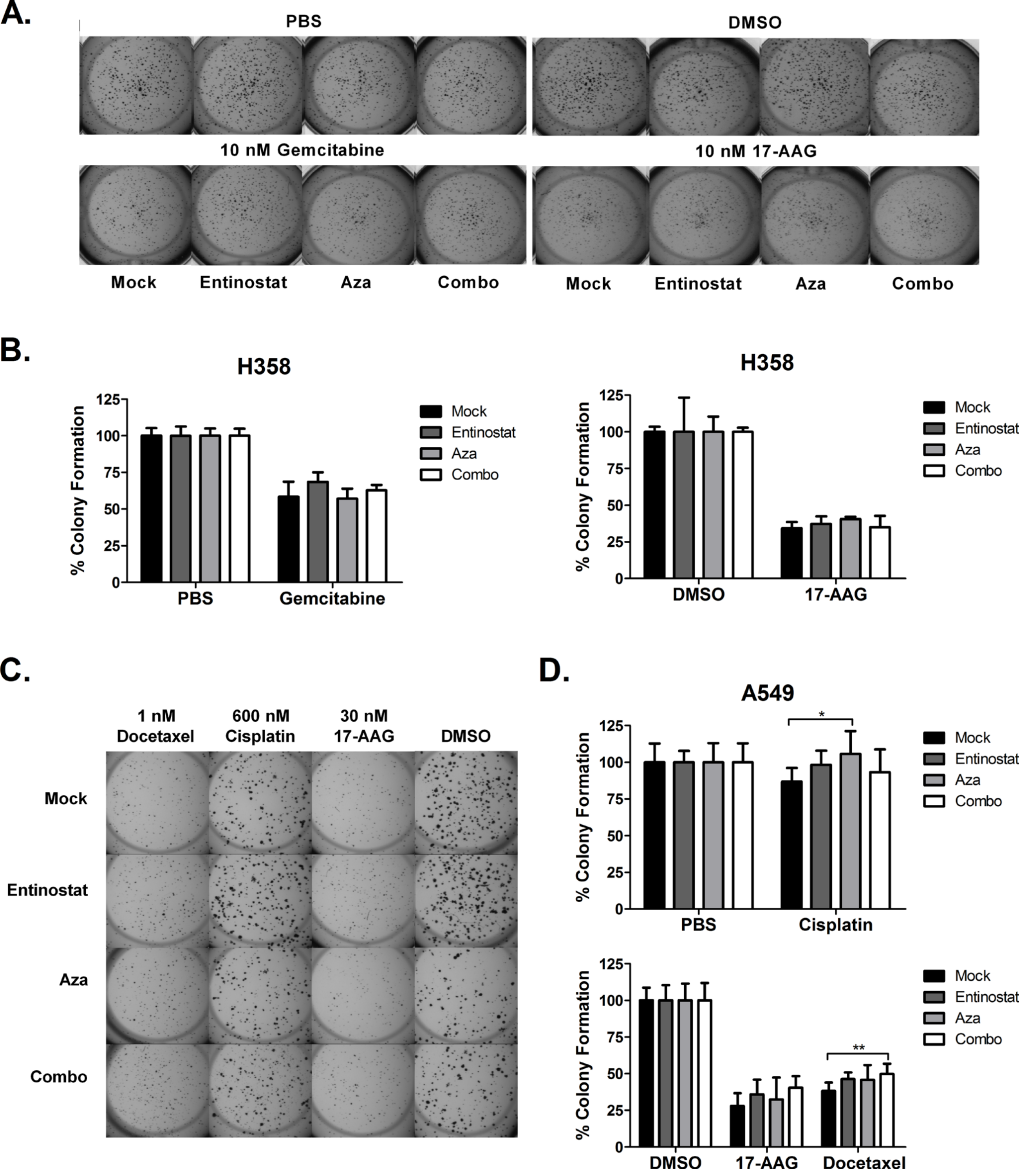
Epigenetic priming does not potentiate the effects chemotherapy on colony growth H358 and A549 cells were seeded on a solidified Matrigel layer six days after epigenetic therapy. Beginning the following day, cells were treated with chemotherapy for 72 hours. Drug was then removed and colonies were permitted to grow 2 – 4 additional days. **(A)** Representative H358 colonies following treatment with 10 nM gemcitabine or 10 nM 17-AAG. **(B)** H358 percent colony formation (+/− standard deviation) relative to untreated control (PBS or DMSO), calculated from one representative experiment with five replicates. **(C)** Representative A549 colonies following treatment with 600 nM cisplatin, 30 nM 17-AAG, or 1 nM docetaxel. **(D)** A549 percent colony formation (+/− standard deviation) relative to untreated control (PBS or DMSO), averaged from two independent experiments (total nine replicates). Statistical significance by ANOVA with Tukey's multiple comparison test denoted as follows: *p < 0.05, **p < 0.01.

H358 and A549 cell lines were selected for their ability to form distinct colonies on a reconstituted basement membrane matrix (Matrigel). Using the treatment paradigm previously described, we pretreated cells with epigenetic therapy. Cells were then seeded at low density on Matrigel one day prior to 72 hour treatment with chemotherapy. Colonies were grown an additional 2 – 4 days after treatment, stained, imaged, and quantified (Fig. 3 and Fig. S4). After normalization to untreated controls within each epigenetic pretreatment group, we found that prior epigenetic treatment did not alter inhibition of H358 colonies by gemcitabine or 17-AAG (Fig. 3A-B and Fig. S4A–B). In A549 cells, pretreatment with azacitidine and combination attenuated colony inhibition by 600 nM cisplatin (−6% vs 13% for mock pretreatment, *p* < 0.05), and 1 nM docetaxel (50% vs 62% for mock pretreatment, *p* < 0.01), respectively (Fig. 3C-D). No significant differences were noted among pretreatment groups in response to 30 nM 17-AAG (Fig. 3C-D), 6 nM bortezomib, and 10 nM 17-AAG (Fig. S4C–D). These data suggest that pretreatment with an inhibitor of DNA methylation and/or histone deacetylation, at the doses tested, does not substantially alter the effects of chemotherapy on anchorage-dependent clonogenic capacity of NSCLC *in vitro*.

### *In vitro* combinatorial epigenetic therapy exerts differential effects on *in vivo* chemosensitivity of NSCLC cell line xenografts

Given the limitations of purely *in vitro* clonogenic model systems, we next assessed whether *in vitro* pretreatment with the combination of azacitidine and entinostat, following the treatment paradigm described above, might alter sensitivity of NSCLC cell line xenografts to subsequent chemotherapy *in vivo*. This model allows for controlled exposure to epigenetic therapy prior to injection of pretreated cells on day 10 to establish xenografts. Two cell lines, A549 and H460, were selected for these studies since prior work in our laboratory demonstrated that their tumor growth rate *in vivo* is unaffected (A549) or only moderately impaired (H460) by epigenetic treatment *in vitro* (data not shown).

**Figure 4:**
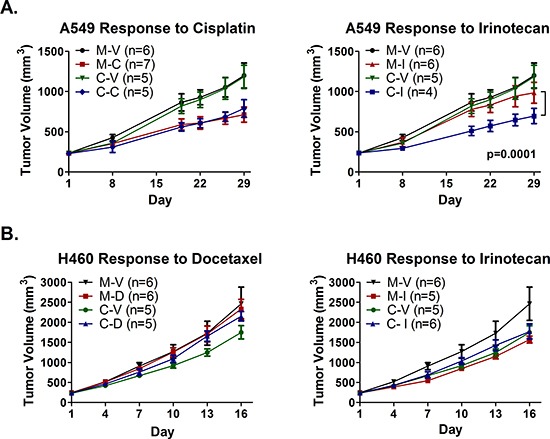
Epigenetic therapy augments response of A549 xenografts, but abrogrates response of H460 xenografts, to irinotecan, and does not sensitize to cisplatin or docetaxel Subcutaneous hind flank tumors were established in NOD/SCID mice from A549 or H460 cells treated *in vitro* with mock (M) or the combination of Aza and entinostat (C). **(A)** Mice bearing pretreated A549 tumors were treated with 2 mg/kg cisplatin (M-C and C-C) on day 2, 10 mg/kg irinotecan (M-I and C-I) on days 2 & 5, or saline vehicle (M-V and C-V), for three one-week cycles. Curves represent mean tumor volume +/− SEM. Statistical significance determined using a mixed effects model and REML. **(B)** Mice bearing pretreated H460 tumors were treated with 2.5 mg/kg docetaxel q4d × 2 escalated to 5 mg/kg docetaxel q4d × 2 (M-D and C-D), 10 mg/kg irinotecan q4d × 4 (M-I and C-I), or saline vehicle (M-V and C-V). Curves represent mean tumor volume +/− SEM.

NOD/SCID mice bearing tumors established from mock and combination epigenetic pretreated A549 cells were randomized to receive vehicle (saline) twice weekly, 2 mg/kg cisplatin weekly, or 10 mg/kg irinotecan twice weekly, over the course of three weeks. Despite a lack of priming to cisplatin in our *in vitro* studies in A549, cisplatin was chosen for this experiment as it is a first-line, standard of care agent for treatment of NSCLC. Irinotecan was included due to the lack of priming to the other chemotherapeutic agents tested in our *in vitro* studies. One animal in the Combo-Irinotecan arm with a tumor more than 3-fold larger in volume than the second largest tumor within that treatment arm, and larger than all tumors in the vehicle control arms for both pretreatments, was considered an outlier and was therefore excluded from subsequent analysis (Fig. S5). We observed no difference in growth between mock and combination pretreated tumors that received vehicle or cisplatin (Fig. 4A). However, epigenetic pretreatment appeared to modestly but consistently augment response to irinotecan compared to mock pretreatment (Fig. 4A). The end of study mean tumor growth inhibition (TGI) following irinotecan treatment was 22% for mock pretreated tumors (M-I) and 51% for combination epigenetic pretreated tumors (C-I), relative to the corresponding vehicle control arms (M-V and C-V, respectively). Irinotecan treatment decreased the rate of growth of M-I and C-I tumors by 5.8 mm^3^/day (*p* = 0.05) and 16.8 mm^3^/day (*p* < 0.0001), respectively, compared to vehicle treatment (M-V and C-V), and the difference in response to irinotecan was statistically significant (*p* = 0.0001). Based on these data, we conclude that *in vitro* pretreatment with combination epigenetic therapy may sensitize A549 xenografts to irinotecan treatment *in vivo*.

Using a similar experimental design, mice bearing tumors established from mock and combination epigenetic pretreated H460 cells were randomized to receive vehicle (saline), 2.5 mg/kg docetaxel, or 10 mg/kg irinotecan, every fourth day for a total of four injections. Docetaxel was selected in place of cisplatin due to existing data that demonstrated synergy between docetaxel and entinostat in H460 *in vitro* [21]. The dose of docetaxel was escalated to 5 mg/kg for the final two injections due to lack of response at the lower dose. Irinotecan was included given the results observed in A549 xenografts. Pretreatment with combination epigenetic therapy alone (C–V) reduced the rate of H460 tumor growth by 42.5 mm^3^/day (*p* = 0.001) relative to mock control (M–V) (Fig. 4B), and resulted in a TGI of 31%. Interestingly, in mice treated with docetaxel, epigenetic pretreated tumors (C–D) exhibited an increased rate of growth (24.7 mm^3^/day greater increase in tumor volume, *p* = 0.0003) compared to vehicle control (C–V), while mock pretreated tumors (M-D) were unaffected by docetaxel treatment (*p* = 0.72) (Fig. 4C). In addition, while irinotecan inhibited growth of mock pretreated tumors (M–I) by 40% compared to vehicle control (M–V), combination epigenetic pretreated tumors (C–I) did not respond to irinotecan treatment (Fig. 4B).

### *In vivo* combinatorial epigenetic therapy does not sensitize H358 xenografts to cisplatin or irinotecan

**Figure 5:**
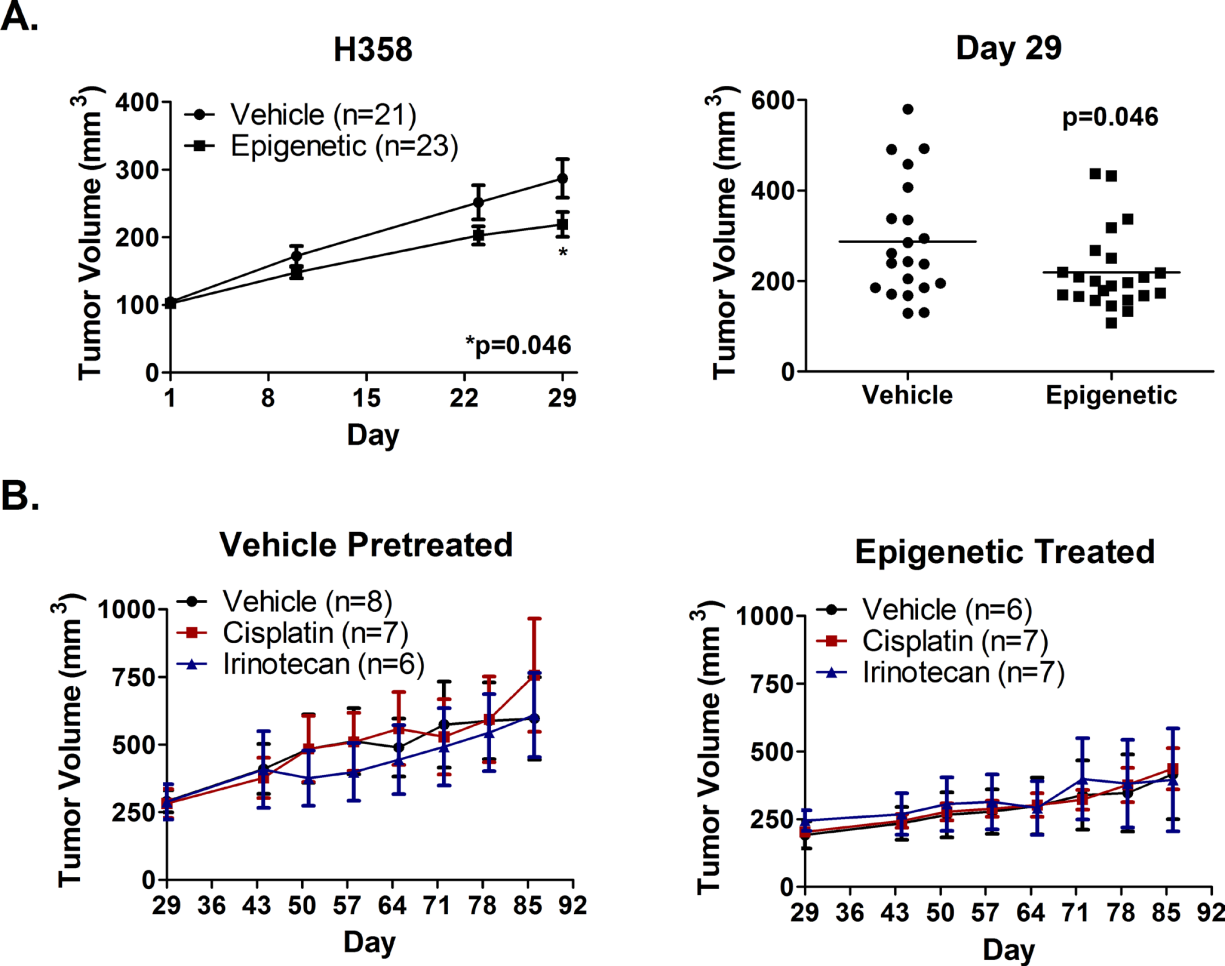
Epigenetic therapy *in vivo* does not sensitize H358 xenografts to immediate subsequent chemotherapy Nude mice bearing H358 xenografts were treated with 0.5 mg/kg Aza (sc, qd × 5) and 1 mg/kg entinostat (ip, day 5), or vehicle, for four one-week cycles. **(A)** Mean tumor volume (+/− SEM) over time (left) and individual tumor volumes at day 29 (right). Statistical significance determined by two-tailed unpaired t test. **(B)** Mean tumor volume (+/− SEM) from vehicle (left) and epigenetic (right) pretreated mice randomized on day 29 to receive 2 mg/kg cisplatin on day 2, 10 mg/kg irinotecan on days 2 & 5, or saline vehicle. Mice were treated for four one-week cycles.

We next assessed whether *in vivo* epigenetic therapy sensitizes NSCLC tumors to immediate subsequent chemotherapy. H358 xenografts were selected due to the slower rate of growth *in vivo*, permitting repeated cycles of epigenetic therapy prior to chemotherapy. Tumor bearing *nu/nu* mice were treated weekly for four weeks with 0.5 mg/kg Aza (sc, days 1 – 5) and 1 mg/kg entinostat (ip, day 5), or with vehicle control, prior to randomization to chemotherapy at the beginning of week five. A similar dose and schedule of entinostat has been used successfully in orthotopic models of NSCLC in rats [32]. The dose of Aza was chosen since it is well tolerated based on prior *in vivo* work, and has been shown to be more efficacious in breast cancer xenografts than higher doses [23]. Following epigenetic therapy, mice were treated with 2 mg/kg cisplatin weekly, 10 mg/kg irinotecan twice weekly, or saline twice weekly for four weeks. Epigenetic therapy inhibited tumor growth by 36% by day 29 (Fig. 5A). However, H358 xenografts showed no evidence of a differential response to subsequent cisplatin or irinotecan therapy, regardless of pretreatment (Fig. 5B).

### Combination epigenetic therapy may prime response to repeat dose irinotecan in a patient-derived NSCLC xenograft model

We extended study of epigenetic priming to a patient-derived xenograft (PDX) model of lung adenocarcinoma, LX7, which has poorly differentiated histology, is negative for *KRAS*, *EGFR* (exons 18–21), and *BRAF* mutations, and is negative for ALK-fusions by cytogenetic analysis. This xenograft was previously established in immunocompromised mice by direct tumor cell transplantation from a NSCLC patient, and is serially passaged in mice. Similarly derived lung cancer PDXs have previously been shown to exhibit gene expression patterns more closely related to primary patient tumors than those of lung cancer cell lines derived from the PDXs [33].

**Figure 6:**
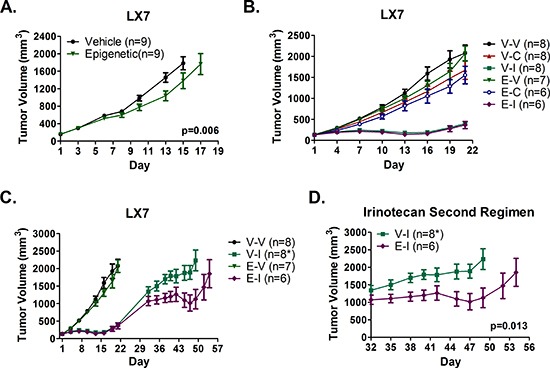
Epigenetic therapy sensitizes a patient derived model of adenocarcinoma to repeat treatment with irinotecan, but does not sensitize to cisplatin **(A)** LX7 bearing NOD/SCID mice were treated with 0.5 mg/kg Aza (sc) on days 1-3, 6-10, 13-17, and 1 mg/kg (ip) entinostat on days 3, 10, 17, or vehicle (*n* = 9 per arm). **(B)** NOD/SCID mice bearing LX7 tumors established from pooled vehicle (V) or epigenetic (E) pretreated tumors were treated with 10 mg/kg irinotecan (V-I and E-I) q4d × 3, 2 mg/kg cisplatin (V-C and E-C) q7d × 2, or saline vehicle (V-V and E-V). **(C-D)** Irinotecan tumors were allowed to grow following cessation of treatment. On day 32, mice were re-challenged with 10 mg/kg irinotecan on days 32, 36, and 40 (same dose and schedule as first cycle). * Two mice in the Vehicle – Irinotecan (V-I) arm were euthanized early (day 42 and 45 after final measurements) for *n* = 6 after day 45. Significance determined using a mixed effects model and REML. All growth curves depict mean tumor volume +/− SEM.

To assess the effects of epigenetic priming on response of LX7 to subsequent chemotherapy, tumor bearing NOD/SCID mice were randomized to receive vehicle or combination epigenetic therapy. Combination epigenetic therapy reduced the tumor growth rate by 19.8 mm^3^/day compared to vehicle (*p* = 0.006), and resulted in a 25% TGI by day 15 (Fig. 6A). Representative tumors from the vehicle and epigenetic arms were harvested days 16 and 17, respectively. Tumors within a given treatment arm were pooled, mechanically processed into a single cell suspension, and frozen for later use.

Mice bearing tumors established from pretreated LX7 tumor cells were randomized to receive 2 mg/kg cisplatin weekly, 10 mg/kg irinotecan every fourth day, or vehicle every fourth day. Treatment consisted of two cisplatin, three vehicle, or three irinotecan intraperitoneal injections. No significant differences in response to cisplatin or irinotecan were observed between vehicle and epigenetic pretreated tumors, with minimal response to cisplatin and tumor regression in response to irinotecan for both pretreatment conditions (Fig. 6B). However, the growth rate of irinotecan treated tumors in both pretreatment arms increased within three days of cessation of irinotecan treatment. Vehicle and cisplatin treated mice were euthanized on day 22 due to large tumor burden, while mice with irinotecan treated tumors continued under observation. By day 32, no significant difference in mean tumor volume (+/− SEM) between pretreatment arms (1345 ± 136 mm^3^ for vehicle pretreated, 1072 ± 131 mm^3^ for epigenetic pretreated) was noted (*p* = 0.19 by two-tailed unpaired t test). Beginning on day 32, mice were subjected to a repeat cycle of irinotecan therapy. Two of eight mice within the vehicle pretreatment arm were euthanized early due to excessive body weight loss (day 42, final tumor volume = 1576 mm^3^) and a large ulcerated tumor (day 45, final tumor volume = 2789 mm^3^). Interestingly, epigenetic pretreated LX7 tumors exhibited increased sensitivity to irinotecan upon repeat exposure, with regression of mean tumor volume to slightly below baseline (day 32) compared to vehicle pretreated tumors, which exhibited only a brief cytostatic response (Fig. 6C-D). After adjusting for the difference in tumors sizes between arms at the initiation of repeat treatment on day 32, we determined that mean tumor volume increased by 9.6 mm^3^/day less in epigenetic treated mice than in vehicle treated mice (23.9 vs 33.5 mm^3^/day, *p* = 0.013). In both arms, tumor growth rapidly increased roughly one week after the final dose of irinotecan, indicating that the degree of response, but not duration, was altered by epigenetic priming.

## DISCUSSION

Our *in vitro* assays generally showed no evidence in support of the epigenetic priming hypothesis, and collectively, our data suggest that the effects of epigenetic pretreatment of NSCLC on response to subsequent chemotherapy may vary with a given epigenetic or chemotherapeutic agent, and by context. In Matrigel colony formation assays, pretreatment of A549 with Aza or Aza plus entinostat appeared to slightly attenuate the effects of cisplatin or docetaxel, respectively. While statistically significant, these differences are modest, and likely do not suggest a clinically relevant shift in chemotherapy responsiveness. In addition, these effects were not observed in our cell viability experiments, or with cisplatin in the more relevant xenograft model. Response of NSCLC xenografts to cisplatin therapy following *in vitro* (A549) and *in vivo* (H358, LX7) pretreatment with combination epigenetic therapy was unaltered. Conversely, in our H460 xenograft model, epigenetic priming abrogated response of H460 xenografts to irinotecan, and in fact resulted in a slightly increased rate of tumor growth in the docetaxel treated group compared to the vehicle treated group, suggesting the potential for epigenetic priming to negatively impact response to subsequent chemotherapy, at least in select contexts. Mechanistic studies will be important to determine underlying genetic or epigenetic factors responsible for these negative interactions in our model, and what, if any, impact these findings may have on the clinical application of epigenetic priming.

Some evidence in support of an epigenetic priming of chemotherapy response was observed in A549 and LX7 xenografts. Xenografts established from mock pretreated A549 cells were minimally responsive to irinotecan, whereas tumors grown from cells pretreated with combination epigenetic therapy exhibited increased sensitivity to the same irinotecan therapy. Interestingly, in addition to the differential responsiveness of H460 and A549 epigenetic primed tumors to irinotecan, we noted differential effects of the priming therapy in A549 and H460 cells. We saw no change in histone H4 acetylation following combination epigenetic therapy in H460 cells, whereas H4 acetylation increased approximately 5-fold in A549 cells treated with the combination of Aza and entinostat. It is possible that histone hyperacetylation, which appeared to be lacking in H460 primed cells, is a key component of the priming effect observed in A549 xenografts.

The A549 xenograft data are notable given that transient (72 h) *in vitro* treatment with epigenetic therapy did not affect the growth of A549 xenografts, yet altered the response of established xenografts to a chemotherapeutic agent weeks later. This is of interest in light of the clinical observations of several patients whose disease progressed on epigenetic therapy but who experienced better than expected responses to subsequent chemotherapy. These data together with the clinical trial observations suggest that the effects of epigenetic therapy on tumor cells can be long lasting, and that immediate response may not be necessary for patients to derive benefit from this approach.

Some additional evidence in support of an epigenetic priming of chemotherapy response was observed in LX7 xenografts. LX7 PDXs established from vehicle and epigenetically pretreated LX7 tumors both responded strongly to initial, short duration (three injections over nine days) irinotecan therapy, but recovered following cessation of treatment. Upon treatment with a second regimen of irinotecan, epigenetic pretreated LX7 tumors exhibited increased response to therapy, characterized by a slowed growth and regression of mean tumor volume to slightly below baseline (day 32). In comparison, vehicle pretreated tumors exhibited continued tumor growth, with a minor cytostatic effect but no regression. The LX7 data are particularly intriguing for two reasons. First, LX7 is a patient-derived adenocarcinoma model maintained in mice and may better reflect the biology of disease than *ex vivo* cell line models, as suggested by earlier data from our group [33]. Second, LX7 tumors responded well to initial irinotecan therapy, but grew quickly after treatment ended, which is reminiscent of the short-lived response to chemotherapy seen in many NSCLC patients. Tumors that had no prior exposure to epigenetic therapy did not respond as well to the second cycle of irinotecan therapy, as is often the case for recurrent NSCLC, while tumors previously exposed to epigenetic therapy again exhibited at least transient tumor regression.

Data from two models of human lung adenocarcinoma, the cell line A549 and the patient-derived primary xenograft LX7, offer support for the idea that prior exposure to epigenetic therapy may enhance response of established tumors to irinotecan. Balanced against this, data from other models, and from these same models treated with other chemotherapeutic agents, show no evidence of enhanced response with epigenetic priming, and in one case suggest a negative interaction. The effects of prior exposure to epigenetically targeted agents appear to be highly context dependent.

In the majority of models and with the majority of cytotoxic chemotherapy agents tested, we were not able to demonstrate that combinatorial epigenetic therapy with azacitidine and entinostat enhanced tumor sensitivity to subsequent chemotherapy. Taken together, these data call into question whether in fact azacitidine and entinostat exposure could augment chemotherapeutic efficacy in a substantial fraction of lung cancer patients. However, we cannot exclude the possibility that our model systems do not fully reflect the genetic and/or epigenetic landscape of a subset of patients that may derive benefit from epigenetic priming. In addition, there are many potentially relevant differences between efficacy in preclinical models and clinical outcome in patients, notably differences in drug pharmacokinetics. A randomized phase II clinical trial to specifically address this question in patients with advanced lung cancer has been initiated, including priming of irinotecan. A second trial assessing epigenetic priming of an irinotecan-based regimen (FOLFIRI) in colon cancer is also underway. These studies offer the opportunity to address this clinical hypothesis directly. Comparative data from the clinical lung cancer study in progress and the preclinical work presented here, in particular, will be of interest in defining the extent to which these preclinical models can reflect treatment paradigms of relevance to lung cancer patients.

## METHODS

### Cell lines

NCI-H358 and A549 were purchased in from the American Type Culture Collection (ATCC) and were reauthenticated by STR profiling (Promega StemEltite™) immediately prior to use for this work. NCI-H1299, NCI-H838, NCI-H460 were purchased from ATCC immediately prior to use for this work. Experiments were conducted on low passage cells. Cells were routinely checked for mycoplasma (Lonza MycoAlert™ Mycoplasma Detection Kit). Cells were cultured in RPMI-1640 supplemented with 10% FBS, penicillin/streptomycin, 2 mM l-glutamine, 1 mM sodium pyruvate, 10 mM HEPES buffer, and 1.5% sodium bicarbonate, in a humidified incubator at 37ºC with 5% CO_2_.

### Drugs and reagents

5-azacitidine (Aza) was purchased from Tocris Bioscience. For *in vitro* use, Aza was dissolved in 1x PBS as a 4 mM stock and stored at −80ºC in aliquots. Fresh aliquots were thawed immediately prior to use. For *in vivo* studies, Aza was dissolved in saline (0.9% sodium chloride) at 1 mg/mL and stored at −80ºC in aliquots. Fresh aliquots were thawed and diluted 1:10 in saline immediately prior to injection. Entinostat was provided by Syndax Pharmaceuticals. For *in vitro* experiments, entinostat was dissolved in DMSO, diluted to a 200 uM solution, and stored at −20ºC. Drug was thawed immediately prior to use and diluted 1:4000 in culture medium to provide a final concentration of 50 nM entinostat and 0.025% DMSO. For *in vivo* use, entinostat was dissolved at 2 mg/ml in DMSO and stored at −20ºC. Immediately prior to injection, entinostat was thawed and diluted 1:10 in saline. 17-AAG and bortezomib, and docetaxel and vinorelbine were purchased from LC Labs and Selleck Chemicals, respectively, and dissolved in DMSO for *in vitro* use. Appropriate dilutions in DMSO were made to provide a final of 0.025% DMSO in medium. Cisplatin (APP Pharmaceuticals), Gemcitabine (Sagent Pharmaceuticals), Docetaxel (Hospira), and Camptosar (Pfizer) were obtained from the Johns Hopkins Hospital pharmacy. Gemcitabine was dissolved in saline. All drugs were diluted with saline for *in vivo* use. Cisplatin and gemcitabine were diluted with 1x PBS for *in vitro* use.

### Treatment of cell lines with epigenetic therapy

Cells were seeded at the following densities (per 75 cm^2^): 2 × 10^5^ (H1299, H460), 3.5 × 10^5^ (H838, A549), 1 × 10^6^ (H358). Cells were allowed to adhere 20-24 h and were treated with 500 nM Aza or mock (1x PBS) in fresh media every 24 h for 48 h, followed by treatment with 500 nM Aza, 50 nM entinostat, combination, or mock in fresh media (final 0.025% DMSO for all treatments) for an additional 24 h. After the 72 h treatment, cells harvested and reseeded at equal density and cultured in drug-free media.

### DNA methylation analysis

DNA from mock, entinostat, Aza, or combination treated cells was harvested at the end of treatment (day 3) and one week post treatment (day 10). Global methylation analysis was performed on bisulfite converted DNA samples using the Illumina Infinium HumanMethylation450 BeadChip (Illumina, Inc.). Data were processed using R and Bioconductor. Box plots were generated using deltaBeta values (deltaBeta = treatment Beta value – mock Beta value) for probes in which mock Beta value was >0.5.

### HDAC activity assay

HDAC2 enzyme activity was assessed in cells treated for 24 h with 50 nM entinostat or mock using the HDAC-Glo 2 Assay (Promega), nonlytic format, and quantified on a Safire^2^ plate reader (Tecan). Raw data were corrected for background luminescence and normalized to mock to determine relative HDAC2 activity.

### Western blot for histone H4 acetylation

Following epigenetic treatment as described above, cells were washed and lysed to isolate intact nuclei. Collected nuclei were washed and lysed and the insoluble histone containing fraction was retained. Sodium butyrate (5 mM) was added to all wash and lysis buffers to retain existing histone acetylation. The histone fraction was nuclease treated with benzonase (250 units), and diluted in an equal volume of 2x Laemmli sample buffer (Bio-Rad). Additional details are provided in the Supplementary Methods. Western blotting was performed using standard techniques and the following primary antibodies from Millipore: Anti-acetyl Histone H4 (Lys5/8/12/16), clone 3HH4-4C10, 1:1000 dilution, and Anti-Histone H4, pan, clone 62-10C-2, 1:2000 dilution. Following detection of acetyl histone H4, blots were stripped for 45 min at room temperature in Restore stripping buffer (Thermo Scientific) and re-probed for total histone H4. Protein levels were quantified using ImageJ software.

### Cell viability assays

Six days after epigenetic treatment (day 9), pretreated cells were seeded in triplicate in white walled 96-well plates as follows: H1299 = 1000, H358 = 4200 H838 = 1700, and A549 = 1500 cells/well. Approximately 24 h later, cells were treated for 72 h with 17-AAG (3 – 1000 nM, 0.03 – 10 uM for H358), bortezomib (1 – 300 nM), cisplatin (0.03 – 10 uM), docetaxel (0.3 – 100 nM, 0.3 – 30 nM for A549), gemcitabine (1-300 nM), or vinorelbine (0.3 – 100 nM). Cell viability was assessed using the CellTiter-Glo Luminescent Cell Viability Assay (Promega) and quantified on a SpectraMax M2e plate reader (Molecular Devices). Raw data were corrected for background luminescence, transformed (x=log(x)), and analyzed by nonlinear regression (log(inhibitor) vs. response with variable slope) in GraphPad Prism 5 to obtain IC_50_ values, 95% confidence intervals, and R^2^. IC_50_ was considered not determined if calculated as ambiguous by Prism. Transformed data were then normalized to untreated controls within each pretreatment group for a given chemotherapy to generate log dose response curves. Experiments were repeated at least twice to ensure consistent results, with the exception of vinorelbine in H358 and H1299, as no differences were observed in any cell line. Results from representative experiments are shown.

### Matrigel colony formation assays

Six days after epigenetic treatment (day 9), pretreated H358 and A549 cells were seeded at low density in 96-well plates (4-5 replicates) on a 40 uL layer of solidified Matrigel (Corning). Approximately 24 h later, cells were treated with 10 nM gemcitabine, 10 nM 17-AAG, or 20 nM 17-AAG (H358) or 600 nM cisplatin, 6 nM bortezmib, 10 nM 17-AAG, 30 nM 17-AACG, or 1 nM docetaxel (A549). After 72 h, chemotherapy was removed, cells were rinsed with 1x PBS, and drug free media was added. Colonies were grown an additional 2 – 4 days, stained with MTT reagent, and imaged and counted on the GelCount™ colony counter (Oxord Optronix). Colony number was normalized to untreated control within a given pretreatment group to determine percent colony formation after chemotherapy.

### Animal xenografts studies

Protocols for animal experiments were approved by the John Hopkins University Animal Care and Use Committee and were strictly followed. Female NOD/SCID mice were obtained from the Johns Hopkins Research Animal Resources. Female nude (*Foxn1^nu/nu^*) mice were purchased from Jackson Laboratory. All xenografts were established from cells injected subcutaneously into the right hind flank of 6 – 7 week old female mice, in a total volume of 100 uL, consisting of a 1:1 mix of 1x PBS and Matrigel. Tumor volume was calculated as (L × W^2^)/2. Azacitidine was administered subcutaneously at 0.5 mg/kg. Entinostat was administered intraperitoneally at 1 mg/kg. All other chemotherapeutics were administered intraperitoneally. Vehicle for entinostat was 10% DMSO in saline. All other vehicle injections were saline. Injection volume was 5 mL/kg. Mean tumor growth inhibition was calculated as TGI = (1-(T_f_-T_0_)/(C_f_-C_0_))*100, where T_f_ and T_0_ represent final and initial mean tumor volumes in the treatment arm, respectively, and C_f_ and C_0_ represent final and initial mean tumor volumes in the vehicle control arm, respectively.

### Xenografts established from pretreated cells

Seven days after epigenetic treatment (day 10), mock and combination pretreated A549 and H460 cells were harvested and counted, and 6.5 × 10^5^ (A549) and 2.75 × 10^5^ (H460) viable cells/mouse were injected into NOD/SCID mice. Mice were added to study when tumors reached 250 mm^3^ (+/− 20%). A549 bearing mice were treated with 2 mg/kg cisplatin (days 2, 9, 16), 10 mg/kg irinotecan (days 2, 5, 9, 12, 16, 19), or vehicle (days 2, 5, 9, 12, 16, 19). H460 bearing mice were treated with 2.5 mg/kg docetaxel (q4d × 2) escalated to 5 mg/kg docetaxel (q4d × 2), 10 mg/kg irinotecan (q4d × 4) or vehicle, starting on day 1.

### H358 xenografts and therapeutic administration of epigenetic therapy

Untreated H358 cells were injected into female nude mice (8 × 10^5^ cells/mouse) and tumors were grown to a starting size of 110 mm3 (+/− 25%). Mice were treated each week with 0.5 mg/kg Aza (days 1 – 5) and 1 mg/kg entinostat (day 5), or vehicle, for four one-week cycles. Beginning week five, mice received 2 mg/kg cisplatin (day 2), 10 mg/kg irinotecan (days 2 & 5), or vehicle (days 2 & 5), weekly for four weeks.

### Patient-derived primary xenografts experiments

Untreated LX7 cells collected from freshly grown tumors were injected into NOD/SCID mice (10^6^ cells/mouse). Tumors were grown to a starting size of approximately 160 mm^3^ (+/− 25%). Mice were randomized to receive vehicle or azacitidine (days 1 – 3, 6 – 10, 13 – 17) and entinostat (days 3, 10, 17). On day 16 and 17, representative vehicle and epigenetic treated tumors, respectively, were harvested and pooled. Single cell suspensions were generated and frozen at −80°C in 90% FBS/10% DMSO. Cells were later thawed, and 1.3 × 10^6^ cells/mouse were injected into NOD/SCID mice. Once tumors reached approximately 140 mm^3^ (+/− 15%), mice were treated with 2 mg/kg cisplatin (q7d × 2), 10 mg/kg irinotecan (q4d × 3, repeated on day 32), or vehicle (q4d × 3).

### Statistical analysis

ANOVA with Tukey's multiple comparison test and two-tailed unpaired t test were performed in Graphpad Prism 5. For A549, H460, and LX7 xenograft experiments. Linear mixed effects model with random intercept was used to fit longitudinal tumor data. Group by time interactions were used to compare tumor growth rates between groups. All model parameters were estimated through restricted maximum likelihood (REML) methods.

## SUPPLEMENTARY METHODS, FIGURES AND TABLE


