# Supplementary material for: Evaluation of azacitidine and entinostat as sensitization agents to cytotoxic chemotherapy in preclinical models of non-small cell lung cancer
Source: Oncotarget. 2014 Nov 25;6(1):56–70. doi: 10.18632/oncotarget.2695 (PMC4381578; doi:10.18632/oncotarget.2695)
Supplement: Supplementary file 1 [file oncotarget-06-056-s001.pdf]

# SUPPLEMENTARY METHODS, FIGURES AND TABLE

## Cell fractionation and sample preparation for Western Blot

Adherent cells were washed with 1x PBS containing 5 mM sodium butyrate (NaB) and directly lysed in 10 mM Tris-HCl (pH 7.9), 0.34 M sucrose, 3 mM CaCl2, 2 mM magnesium acetate, 0.1 mM EDTA, 0.5% Nonidet P-40, protease inhibitors, and 5 mM NaB for 15 min on ice to isolate nuclei. Nuclei were collected by centrifugation (1500 x g for 15 min at 4°C), washed in cytoplasmic lysis buffer without Nonidet P-40, and lysed in 20 mM HEPES (pH 7.9), 3 mM EDTA, 10% glycerol, 150 mM potassium acetate, 1.5 mM MgCl2, 0.1% Nonidet P-40, protease inhibitors, and 5 mM NaB. The insoluble histone-containing pellet was retained and nuclease treated in 150 mM HEPES (pH 7.9), 10% glycerol, 150 mM potassium acetate, 1.5 mM MgCl2, protease inhibitors, and benzonase (250 units) for 10 min at 37°C. After nuclease incubation, an equal volume of 2x Laemmli sample buffer (Bio-Rad) was added. Immediately prior to gel loading, β-mercaptoethanol was added and samples were heated at 95°C for 5 min to reduce and denature proteins. Samples were electrophoresed on NuPage 12% Bis-Tris gels with 1 × MOPS running buffer (Novex, Life Technologies), transferred to 0.2 µm pore PVDF membranes.

## SUPPLEMENTARY FIGURES AND TABLE

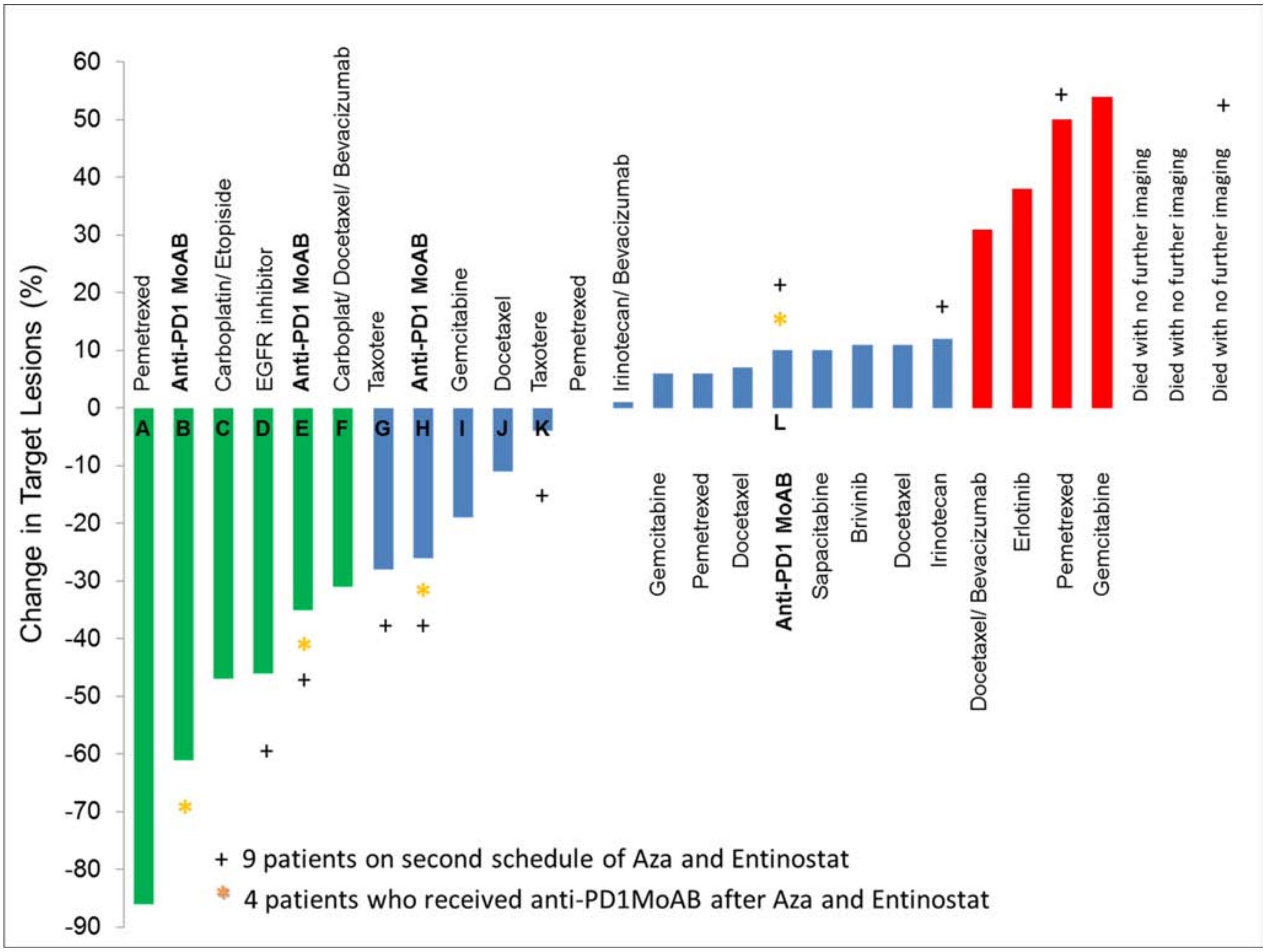


**Supplementary Figure S1: Maximal change in tumor size in response to subsequent chemotherapy following combinatorial epigenetic therapy.** Green bars represent objective responses by RECIST criteria to specified subsequent treatment regimen as measured by the percent change in maximal diameters of target lesions. Blue bars represent disease stabilization, while red bars indicate disease progression. Graphs updated from Juergens et al., 2011.

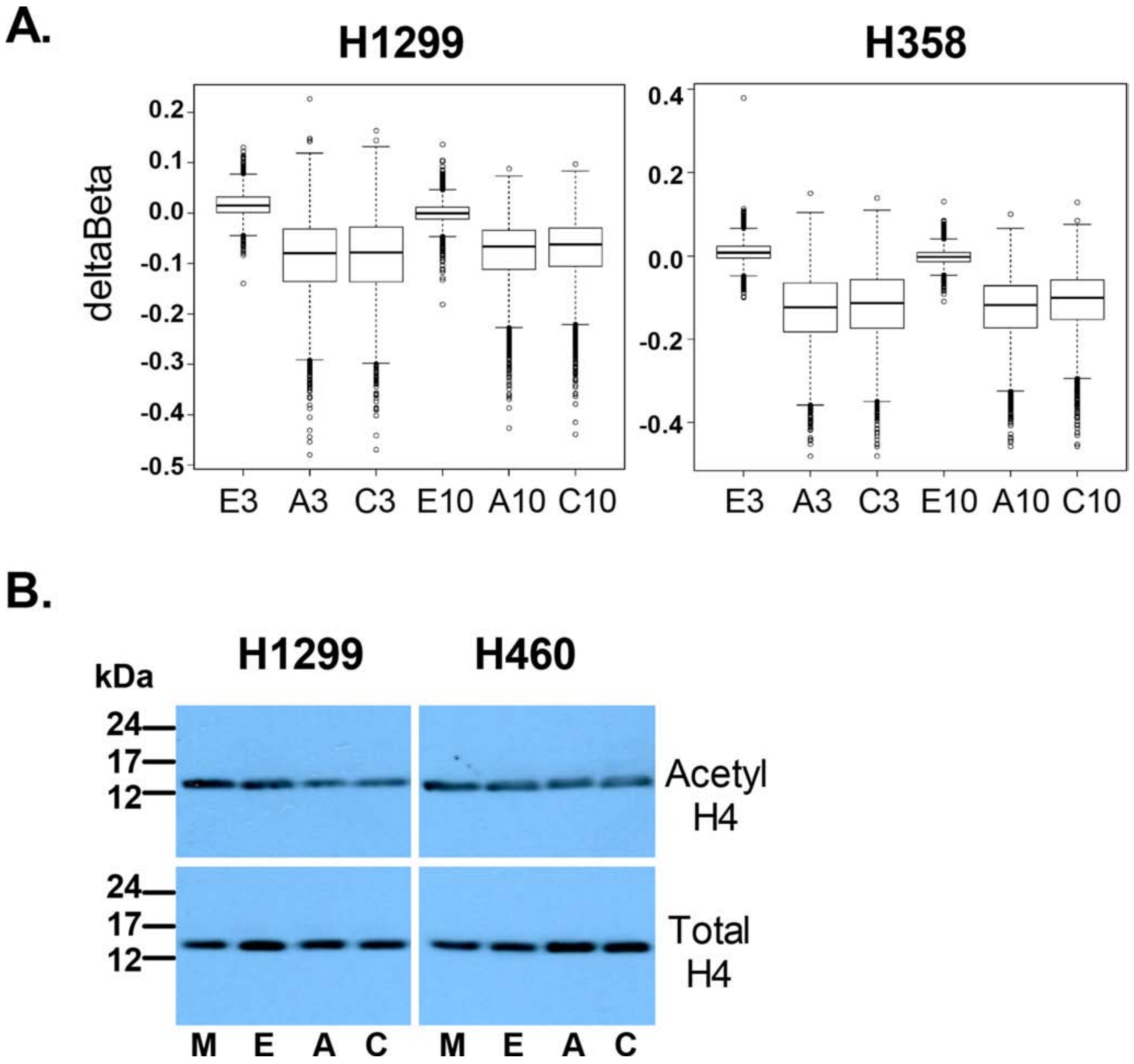


**Supplementary Figure S2: Epigenetic changes associated with azacitidine and entinostat treatment. (A)** Box plots of deltaBeta values depicting promoter region (+/− 1500 bp of transcription start site) demethylation (negative deltaBeta) relative to mock control (probes with Beta >0.5) at day 3 and day 10 following treatment with entinostat (E), Aza (A), or combo (C). **(B)** Western blots depicting acetylated histone H4 (lysine 5, 8, 12, 16) and total histone H4 levels at the end of treatment (day 3) with mock (M), entinostat (E), Aza (A), or combo (C).

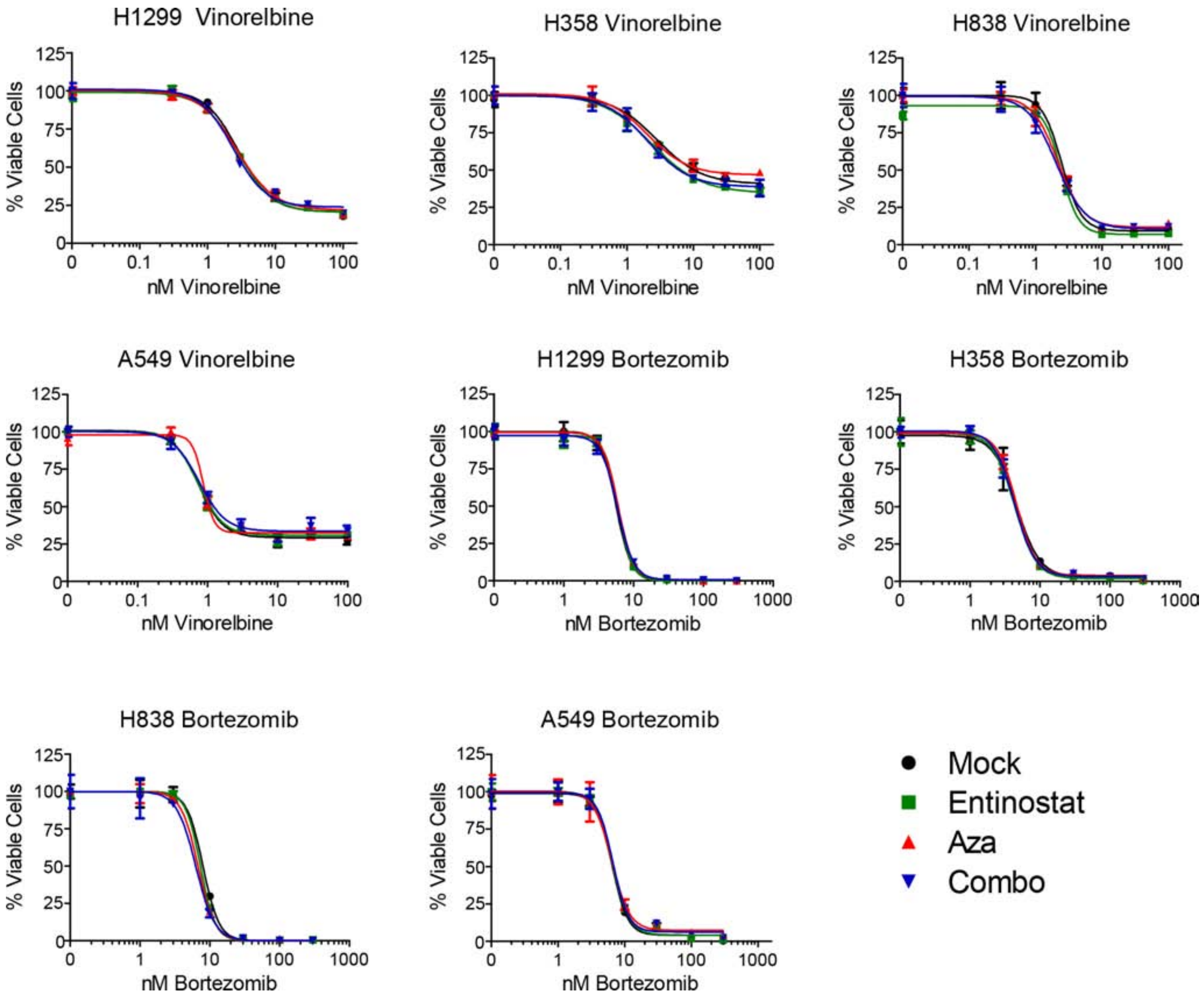


**Supplementary Figure S3: Epigenetic priming does not alter chemosensitivity of NSCLC cell lines.** Log dose response curves for NSCLC cell lines treated with bortezomib or vinorelbine for 72 hours one week post epigenetic therapy. Individual curves represent the percentage of viable cells (+/− standard deviation) for each epigenetic pretreatment condition normalized to its own untreated control cells, such that the highest values for each pretreatment condition represent 100%, and 0 = 0%. Data shown from representative experiments.

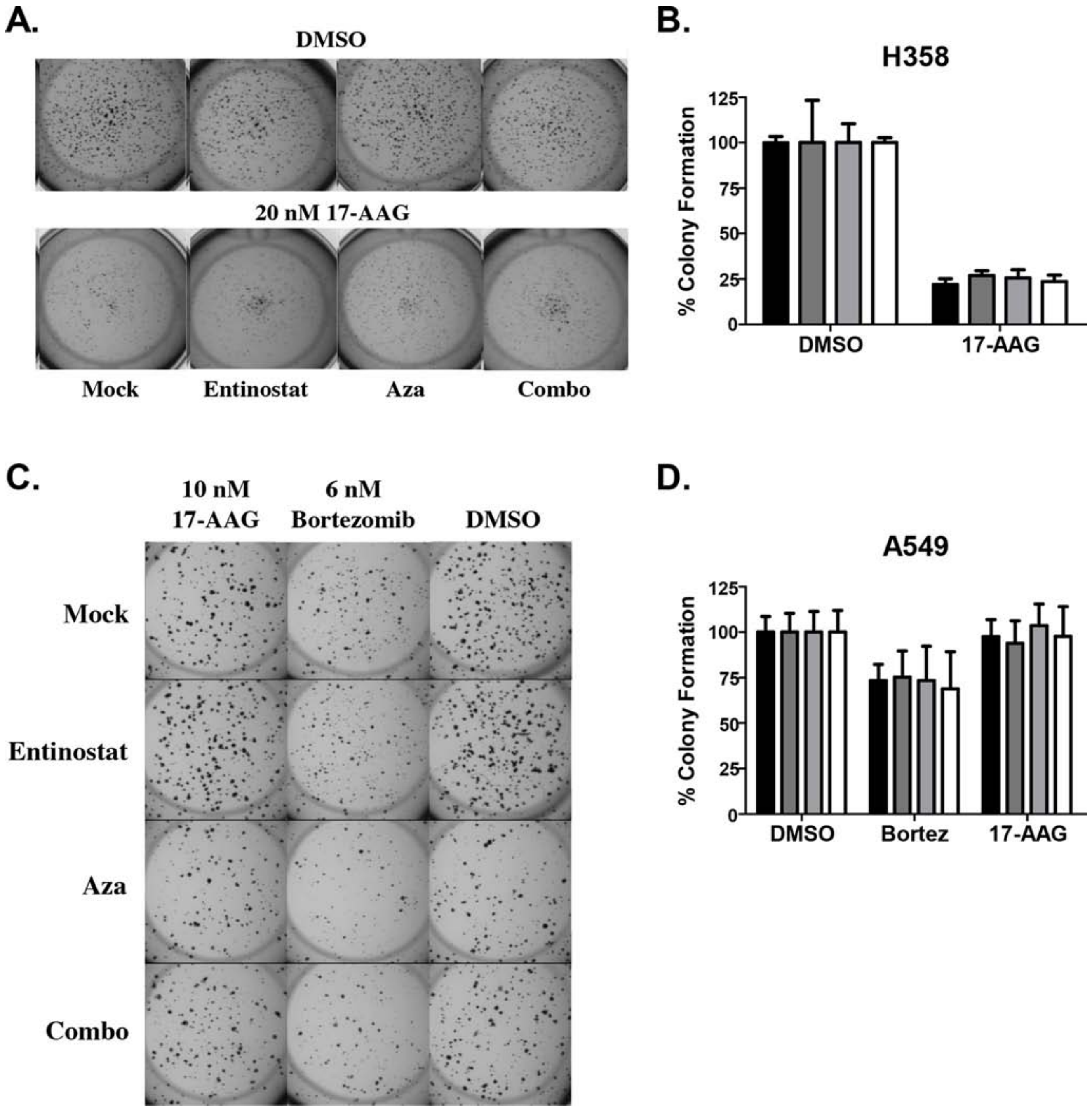


**Supplementary Figure S4: Epigenetic therapy does not potentiate the effects of chemotherapy on colony growth on Matrigel.** H358 and A549 cells were seeded on a solidified Matrigel layer six days after epigenetic therapy. The following day, cells were treated with chemotherapy for 72 hours. Drug was then removed and colonies were permitted to grow 2 – 4 additional days. **(A)** Representative H358 colonies following treatment with 20 nM 17-AAG. **(B)** H358 percent colony formation (+/− standard deviation) relative to untreated control (DMSO), calculated from one representative experiment with five replicates. **(C)** Representative A549 colonies following treatment with 6 nM bortezomib or 10 nM 17-AAG. **(D)** A549 percent colony formation (+/− standard deviation) relative to untreated control (DMSO), averaged from two independent experiments (total nine replicates). Statistical significance by ANOVA with Tukey's multiple comparison test denoted as follows: *$p < 0.05$, **$p < 0.01$.

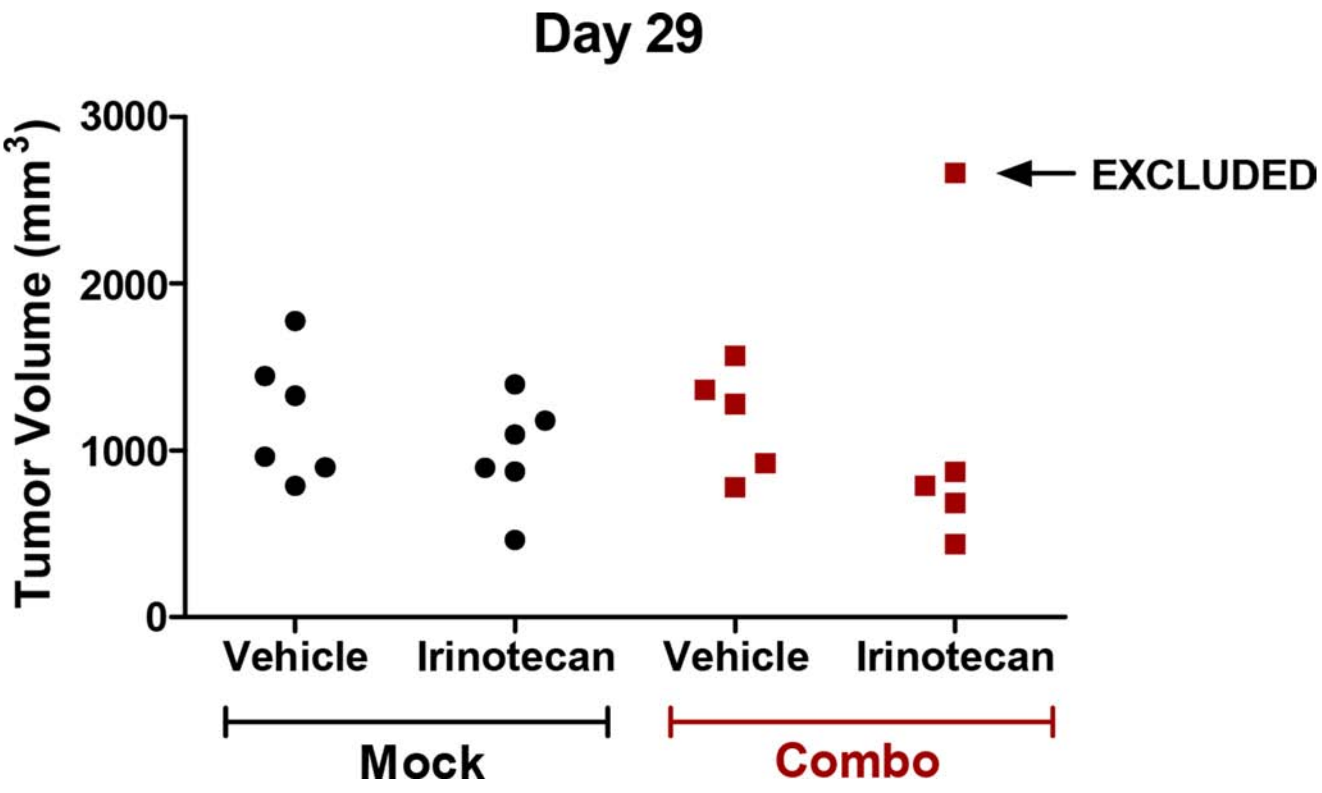


**Supplementary Figure S5: Response of A549 xenografts to irinotecan is augmented by epigenetic therapy.** NOD/SCID mice bearing subcutaneous hind flank tumors established from A549 cells treated *in vitro* with mock or the combination of Aza and entinostat (combo) were randomized to receive 10 mg/kg irinotecan (days 2 & 5) or saline vehicle for three one-week cycles. Individual volumes for each animal at day 29 are shown. One tumor was deemed an outlier and excluded from subsequent statistical analysis.

**Supplementary Table S1. Calculated $IC_{50}$ values for chemotherapy following epigenetic priming.** $IC_{50}$, 95% CI, and $R^2$ calculated from representative experiments with three replicates per dose tested. ND denotes $IC_{50}$ values were not determined.

| Cell Line | Condition | IC50 | 95% CI | $R^2$ |
|---|---|---|---|---|
| **Cisplatin** | | | | |
| **H1299** | Mock | 1.78 µM | 1.40, 2.26 | 0.971 |
| | Entinostat | 1.72 µM | 1.21, 2.44 | 0.964 |
| | Aza | 1.40 µM | 1.10, 1.77 | 0.980 |
| | Combination | 1.53 µM | 0.93, 2.51 | 0.940 |
| **H358** | - | ND | - | - |
| **H838** | - | ND | - | - |
| **A549** | Mock | 2.98 µM | 2.34, 3.80 | 0.972 |
| | Entinostat | 3.35 µM | 1.83, 6.13 | 0.962 |
| | Aza | 3.60 µM | 1.64, 7.89 | 0.878 |
| | Combination | 4.21 µM | 0.92, 19.36 | 0.911 |
| **Docetaxel** | | | | |
| **H1299** | Mock | 2.35 nM | 1.90, 2.89 | 0.986 |
| | Entinostat | 2.30 nM | 1.91, 2.78 | 0.986 |
| | Aza | 2.16 nM | 1.81, 2.59 | 0.989 |
| | Combination | 2.40 nM | 2.11, 2.72 | 0.992 |
| **H838** | Mock | 1.38 nM | 1.15, 1.65 | 0.986 |
| | Entinostat | 1.12 nM | 1.01, 1.24 | 0.995 |
| | Aza | 1.26 nM | 1.10, 1.44 | 0.992 |
| | Combination | 1.07 nM | 0.77, 1.49 | 0.962 |
| **A549** | Mock | 1.12 nM | 0.74, 1.69 | 0.970 |
| | Entinostat | 0.99 nM | 0.74, 1.32 | 0.982 |
| | Aza | 1.50 nM | 1.09, 2.07 | 0.981 |
| | Combination | 1.08 nM | 0.69, 1.69 | 0.967 |
| **Gemcitabine** | | | | |
| **H1299** | Mock | 7.74 nM | 6.70, 8.94 | 0.989 |
| | Entinostat | 6.94 nM | 5.89, 8.17 | 0.989 |
| | Aza | 5.88 nM | 4.38, 7.87 | 0.966 |
| | Combination | 6.08 nM | 5.02, 7.34 | 0.985 |
| **H838** | Mock | 38.55 nM | 26.08, 56.98 | 0.927 |
| | Entinostat | 42.75 nM | 31.31, 58.38 | 0.958 |
| | Aza | 38.94 nM | 27.84, 54.45 | 0.946 |
| | Combination | 44.80 nM | 31.63, 63.45 | 0.951 |

(*Continued*)

| Cell Line | Condition | IC50 | 95% CI | $R^2$ |
|---|---|---|---|---|
| **Vinorelbine** | | | | |
| **H1299** | Mock | 2.84 nM | 2.55, 3.17 | 0.994 |
| | Entinostat | 2.78 nM | 2.48, 3.12 | 0.994 |
| | Aza | 2.73 nM | 2.40, 3.10 | 0.993 |
| | Combination | 2.39 nM | 2.067, 2.76 | 0.990 |
| **H358** | Mock | 2.84 nM | 2.27, 3.56 | 0.985 |
| | Entinostat | 2.35 nM | 1.87, 2.95 | 0.987 |
| | Aza | 2.03 nM | 1.54, 2.68 | 0.974 |
| | Combination | 2.08 nM | 1.55, 2.78 | 0.972 |
| **H838** | Mock | 2.48 nM | 2.21, 2.78 | 0.990 |
| | Entinostat | 2.42 nM | 2.14, 2.74 | 0.990 |
| | Aza | 2.17 nM | 1.93, 2.45 | 0.992 |
| | Combination | 2.03 nM | 1.73, 2.39 | 0.987 |
| **A549** | Mock | 0.74 nM | 0.64, 0.85 | 0.989 |
| | Entinostat | 0.73 nM | 0.64, 0.83 | 0.990 |
| | Aza | 0.87 nM | 0.37, 2.04 | 0.981 |
| | Combination | 0.75 nM | 0.621, 0.91 | 0.979 |
| **17-AAG** | | | | |
| **H1299** | Mock | 99.29 nM | 92.06, 107.1 | 0.995 |
| | Entinostat | 94.44 nM | 86.83, 102.7 | 0.996 |
| | Aza | 93.99 nM | 82.25, 107.4 | 0.991 |
| | Combination | 102.0 nM | 91.60, 113.6 | 0.990 |
| **H358** | Mock | 42.19 nM | 29.45, 60.43 | 0.921 |
| | Entinostat | 31.56 nM | 28.13, 35.41 | 0.985 |
| | Aza | 41.49 nM | 33.84, 50.88 | 0.971 |
| | Combination | 37.57 nM | 33.71, 41.87 | 0.990 |
| **A549** | Mock | 46.38 nM | 37.55, 57.29 | 0.983 |
| | Entinostat | 44.42 nM | 35.82, 55.08 | 0.981 |
| | Aza | 34.28 nM | 28.15, 41.74 | 0.971 |
| | Combination | 37.62 nM | 31.07, 45.56 | 0.990 |
| **Bortezomib** | | | | |
| **H1299** | Mock | 5.62 nM | 5.06, 6.24 | 0.997 |
| | Entinostat | 6.00 nM | 5.25, 6.86 | 0.996 |
| | Aza | 6.00 nM | 5.71, 6.29 | 0.999 |
| | Combination | 5.87 nM | 5.27, 6.52 | 0.996 |

(*Continued*)

| Cell Line | Condition | IC50 | 95% CI | $R^2$ |
|---|---|---|---|---|
| **H358** | Mock | 4.65 nM | 3.88, 5.56 | 0.983 |
| | Entinostat | 4.34 nM | 3.88, 4.86 | 0.993 |
| | Aza | 4.62 nM | 4.31, 4.96 | 0.998 |
| | Combination | 4.33 nM | 3.98, 4.71 | 0.996 |
| **H838** | Mock | 8.08 nM | 6.89, 9.47 | 0.994 |
| | Entinostat | 7.58 nM | 6.84, 8.40 | 0.999 |
| | Aza | 6.80 nM | 6.18, 7.48 | 0.997 |
| | Combination | 6.49 nM | 5.30, 7.95 | 0.985 |
| **A549** | Mock | 6.40 nM | 5.54, 7.40 | 0.993 |
| | Entinostat | 6.41 nM | 5.67, 7.26 | 0.994 |
| | Aza | 6.38 nM | 5.06, 8.03 | 0.978 |
| | Combination | 6.62 nM | 5.40, 8.12 | 0.987 |
